# Presence and Persistence of Viable, Clinically Relevant Legionella pneumophila Bacteria in Garden Soil in the Netherlands

**DOI:** 10.1128/AEM.00595-16

**Published:** 2016-08-15

**Authors:** E. van Heijnsbergen, A. van Deursen, M. Bouwknegt, J. P. Bruin, A. M. de Roda Husman, J. A. C. Schalk

**Affiliations:** a National Institute for Public Health and the Environment, Bilthoven, the Netherlands; b Regional Public Health Laboratory Kennemerland, Haarlem, the Netherlands; c Institute for Risk Assessment Sciences, Utrecht University, Utrecht, the Netherlands; Washington State University

## Abstract

Garden soils were investigated as reservoirs and potential sources of pathogenic Legionella bacteria. Legionella bacteria were detected in 22 of 177 garden soil samples (12%) by amoebal coculture. Of these 22 Legionella-positive soil samples, seven contained Legionella pneumophila. Several other species were found, including the pathogenic Legionella longbeachae (4 gardens) and Legionella sainthelensi (9 gardens). The L. pneumophila isolates comprised 15 different sequence types (STs), and eight of these STs were previously isolated from patients according to the European Working Group for Legionella Infections (EWGLI) database. Six gardens that were found to be positive for L. pneumophila were resampled after several months, and in three gardens, L. pneumophila was again isolated. One of these gardens was resampled four times throughout the year and was found to be positive for L. pneumophila on all occasions.

**IMPORTANCE** Tracking the source of infection for sporadic cases of Legionnaires' disease (LD) has proven to be hard. L. pneumophila ST47, the sequence type that is most frequently isolated from LD patients in the Netherlands, is rarely found in potential environmental sources. As L. pneumophila ST47 was previously isolated from a garden soil sample during an outbreak investigation, garden soils were investigated as reservoirs and potential sources of pathogenic Legionella bacteria. The detection of viable, clinically relevant Legionella strains indicates that garden soil is a potential source of Legionella bacteria, and future research should assess the public health implication of the presence of L. pneumophila in garden soil.

## INTRODUCTION

*L*egionella is an opportunistic pathogen that can cause legionellosis (1). Legionellosis refers to two distinct clinical syndromes: Legionnaires' disease (LD) and Pontiac fever. The vast majority of the clinical isolates in Europe (2–4) and the United States (5) constitute Legionella pneumophila serogroup 1 (SG1). In Australia, New Zealand, and Thailand, Legionella longbeachae is an important cause of disease (6–8). Legionella bacteria are ubiquitous in natural matrices and manmade water systems (9). Some of these systems and matrices can act as sources of Legionella infection and include cooling towers (10), whirlpools (11), thermal springs (12), and wastewater treatment plants (13). Potting soil is also a source of L. longbeachae infection (14, 15).

In source investigations, alongside epidemiological evidence, a genotypic match between environmental and clinical strains is needed to identify the source of infection. Tracking the source of infection for sporadic cases of LD has proven to be hard (16). Studies in the Netherlands, England, and Wales showed that common clinical L. pneumophila strains are only rarely found in the environment (17, 18). In these studies, L. pneumophila genotypes isolated from patients were compared with genotypes isolated from the environment. The environmental sources of Legionella in these studies comprised manmade water systems (domestic water distribution systems, cooling towers, and spa pools).

L. pneumophila sequence type 47 (ST47) is the ST that is most frequently isolated from patients in the Netherlands (26.9% of clinical isolates available for typing) (16). Strikingly, since the start of the National Legionella Outbreak Detection Programme in the Netherlands in 2002, this ST was only found three times in the environment during outbreak investigations, which concerned outdoor whirlpools that were involved in two combined outbreaks of LD and Pontiac fever and one solitary case of LD. As all three whirlpools were located outside, it was hypothesized that the outdoor environment was an influence, and after further investigation, L. pneumophila ST47 was isolated from a soil sample from the garden of the most recent outbreak (19). It was suggested that the ST47 strain was transmitted from garden soil to the whirlpool by wind or by people entering the whirlpool with soil on their feet.

It is possible that garden soil plays a role in Legionella infection. Although potting soil is a well-studied reservoir and known source of Legionella, not much is known about Legionella in garden soil. Hughes and Steele (20) showed the presence of Legionella in six garden soils that were mixed with composted materials. Furthermore, there is evidence that natural soil is a reservoir and source of Legionella. Wallis and Robinson (21) reported on a LD patient that had worked at a plant nursery in the week prior to illness. A L. pneumophila strain with an indistinguishable genotypic profile, determined by pulsed-field gel electrophoresis (PFGE), was isolated from a field in which the patient had spent time potting plants. In several other studies L. pneumophila strains have been isolated from natural soil (22–25). In two of these studies, some of the obtained sequence types (STs) had previously been detected in cases of LD (22, 23).

The aim of the current study was to investigate garden soil as a reservoir of viable, clinically relevant Legionella bacteria. Garden soils were sampled throughout the year. Furthermore, we studied whether L. pneumophila could persist in soil over time by resampling Legionella-positive gardens after several months. An amoebal coculture method was applied to detect Legionella bacteria in the soil. Amoebal coculture has been proven successful for the isolation of Legionella bacteria in samples, such as soil, with a lot of background flora (26, 27).

## MATERIALS AND METHODS

### Sampling of garden soils.

Garden soil samples were collected over a period of 1 year (February 2014 to January 2015). In order to obtain a large and diverse number of samples, colleagues from the National Institute for Public Health and the Environment and from the Regional Public Health Laboratory Kennemerland were asked to supply soil samples from their own gardens and/or the gardens of friends and family. Samples were to consist of soil from a soil bed at any place within their own garden, irrespective of type of garden, cultivation, or planting. Sterile spoons and jars were provided and were accompanied by an instruction form and short questionnaire. Participants were asked to sample the upper 2 cm of soil at one place in the garden. Upon arrival, the samples were stored at 4°C until analysis. If L. pneumophila was isolated from a garden, then the owners were asked to resample their garden after several months at the same location as the first sampling.

### Pretreatment of the samples.

Prior to analyses, 5 g of each soil sample was resuspended in 5 ml of sterile distilled water. These suspensions were vortexed for approximately 10 s and incubated at room temperature for 1 h. The soil suspensions were vortexed again just before amoebal coculture.

### Amoebal coculture method.

The amoebal coculture method for the detection of Legionella was performed as described previously (27). Briefly, Acanthamoeba castellanii ATCC 30234 (American Type Culture Collection, Manassas, VA, USA) was grown in 75-cm^2^ culture flasks (Corning Inc., New York, NY, USA) with 15 ml of peptone-yeast extract-glucose (PYG) broth at 25°C. Prior to the infection, the PYG broth was removed, and the amoebae were resuspended in 15 ml Page's amoebal saline (PAS) (28). The amoebae suspension was centrifuged at 850 × *g* for 10 min, and the pellet was subsequently resuspended in 15 ml PAS. This washing step was repeated 2 times. Cells were seeded in a 12-well microplate (Corning) at a density of 5 × 10^5^ cells/ml of PAS. In each well, 1 ml of PAS with amoebae was inoculated with 100 μl of sample, and each sample was tested in triplicate (three wells). Thus, amoebae were inoculated with a total of 300 μl of the suspension. By testing 300 μl of the soil suspension, 0.3 g of the soil sample was tested. The theoretical detection limit was therefore 3.3 CFU/g soil.

The amoebal plates were incubated at 32°C. As a negative control, one well with amoebae was not inoculated with a sample. After 3 days of incubation, 100 μl of each suspension was subcultured on a new plate with freshly seeded amoebae. After another 3 days of incubation at 32°C, 100 μl of each well was serially diluted 10-fold in PAS. Of the 10^4^-, 10^5^-, 10^6^-, 10^7^-, and 10^8^-fold dilutions, 100 μl was plated on buffered charcoal yeast extract (BCYE) plates (Oxoid Ltd., Hampshire, United Kingdom). The 10^5^- and 10^6^-fold dilutions were also cultured on glycine-vancomycin-polymyxin B-cycloheximide (GVPC) plates (Oxoid Ltd., Hampshire, United Kingdom). After 4 and 7 days of incubation at 37°C, the BCYE and GVPC plates were inspected for Legionella-like colonies with a stereo microscope (magnification, 40×; Olympus).

### Confirmation and typing of Legionella isolates.

Suspected Legionella colonies were tested for their inability to grow on BCYE medium without cysteine (Oxoid Ltd., Hampshire, United Kingdom). Strains that were unable to grow on the medium without cysteine were further subtyped by polyclonal antisera (L. pneumophila SG1, SG2 to SG14, and Legionella spp.) coupled to latex beads (Legionella latex test; Oxoid Limited, Hampshire, England) and subsequently stored at −70°C. The strains were later identified by matrix-assisted laser desorption ionization–time of flight mass spectrometry (MALDI-TOF MS). Strains that could not be recultured prior to MALDI-TOF MS identification and that showed no clear agglutination response in the latex test were confirmed with a Legionella species-specific PCR targeting the 5S rRNA gene (29). L. pneumophila isolates were genotyped by the standard sequence-based typing (SBT) method of the European Working Group for Legionella Infections (EWGLI) using seven genes (*flaA*, *pilE*, *asd*, *mip*, *mompS*, *proA*, and *neuA*) (30, 31). The SBT profiles were generated using the high-throughput multilocus sequence typing (HiMLST) method that employs next-generation sequencing (32). L. pneumophila SG1 isolates were also subtyped by monoclonal antibody (MAb) subgrouping using the Dresden MAb panel (33).

### Statistical analysis.

Univariate and multivariate logistic regressions were performed as described by Hosmer and Lemeshow (34). Univariate analysis was used to identify potential associations between the positivity of gardens for Legionella and questionnaire and weather variables. The weather variables, i.e., precipitation and ambient temperature related to sampling days, were obtained from the Royal Netherlands Meteorological Institute (KNMI). For each garden, data were selected from the weather station closest to that garden. Continuous variables (garden age and weather characteristics) were categorized into classes of approximately equal size. Variables with a univariate *P* value of ≤0.25 (two-tailed likelihood ratio test) were selected for multivariate logistic regression. Prior to the multivariate analysis, the correlation coefficients of all variable combinations were determined, where a correlation coefficient of 0.25 or lower was considered noninterfering in the multivariate analysis. Subsequently, backward elimination was used until the remaining variables exhibited a significant association with a *P* value of <0.05 (two-tailed likelihood-ratio test). The statistical analyses were performed using SAS software v.9.3 (SAS Institute, Cary, NC, USA).

## RESULTS

### Isolation of Legionella spp. and L. pneumophila from garden soils.

Over 12 months, a total of 177 unique gardens were sampled. Per month, between 10 and 20 samples were submitted by our colleagues, with an average of 15 (see Table 1). In total, 22 of 177 samples (12%) were positive for Legionella spp. Out of these 22 positive samples, seven (32%) contained L. pneumophila (garden samples 1, 4, 5, 8, 13, 18, and 22; see Table 2). Both L. pneumophila SG1 and non-SG1 isolates were obtained, and 15 different STs were identified. In five of these samples, other Legionella species were also detected besides L. pneumophila, namely, L. longbeachae (4 samples), L. sainthelensi (2 samples), Legionella bozemanii (1 sample), and Legionella feeleii (1 sample). In the remaining 15 samples, several Legionella species were detected (Table 2), including L. sainthelensi (7 samples), L. feeleii (2 samples), L. bozemanii (1 sample), Legionella cincinnatiensis (1 sample), Legionella anisa (1 sample), and Legionella wadsworthii (1 sample). For 3 of the 15 samples, Legionella could not be further typed by MALDI-TOF MS because the bacteria could not be recultured.

**TABLE 1:** Numbers of Legionella species-positive and L. pneumophila-positive garden soil samples per month^*a*^

Sampling yr	Sampling mo	Mean temp (°C) (range)	Total precipitation (mm)	No. of samples analyzed	No. of Legionella species-positive samples (no. of L. pneumophila-positive samples)
2014	February	6.5 (3.9–10)	66.4	14	2 (1)
	March	8.4 (4.5–14.7)	25.7	16	1 (0)
	April	12.1 (7.2–17.1)	58.4	16	4 (2)
	May	13.2 (7.9–19.9)	102.0	15	0 (0)
	June	16.2 (13–21.6)	30.3	16	2 (1)
	July	19.8 (15–26.4)	137.1	20	3 (0)
	August	16.1 (12.4–20.5)	149.0	10	2 (1)
	September	15.9 (21.6–19.3)	20.5	16	3 (0)
	October	13.4 (9.9–17.3)	74.9	16	0 (0)
	November	8.2 (2.5–14.8)	46.8	15	2 (1)
	December	4.8 (−1.2–11.5)	99.5	11	2 (0)
2015	January	4.0 (−2.8–9.9)	115.7	12	1 (1)
Total				177	22 (7)

a The mean ambient temperature (°C) per sampling month and the total precipitation (mm) per month are indicated (35).

**TABLE 2:** Detected Legionella species in garden soil samples

Sampling mo and yr	Garden soil sample	Legionella spp.^*a*^	L. pneumophila serogroup(s) (no. colonies analyzed)	Sequence type(s)
February 2014	1	L. pneumophila	1 (9)	84, 477, 863
		L. pneumophila	2–14 (2)	465, 710
		L. longbeachae	NA^*b*^	NA
	2	L. sainthelensi	NA	NA
March 2014	3	L. cincinnatiensis	NA	NA
April 2014	4	L. pneumophila	1 (6)	84, 115, 477, 2028, 2032
		L. pneumophila	2–14 (12)	863, X^*c*^
		L. bozemanii	NA	NA
		L. feeleii	NA	NA
	5	L. pneumophila	2–14 (8)	2025, 2026, X
	6	L. wadsworthii	NA	NA
	7	L. sainthelensi	NA	NA
June 2014	8	L. pneumophila	1 (2)	84, 710
		L. pneumophila	2–14 (1)	462
		L. longbeachae	NA	NA
	9	L. sainthelensi	NA	NA
July 2014	10	L. feeleii	NA	NA
	11	L. sainthelensi	NA	NA
	12	Legionella spp.^*d*^	NA	NA
August 2014	13	L. pneumophila	2–14 (4)	710
		L. longbeachae	NA	NA
		L. sainthelensi	NA	NA
	14	L. sainthelensi	NA	NA
September 2014	15	L. sainthelensi	NA	NA
	16	Legionella spp.^*d*^	NA	NA
	17	Legionella spp.^*d*^	NA	NA
November 2014	18	L. pneumophila	1 (15)	84, 477, 1856, 2022, 2029
		L. pneumophila	2–14 (3)	84, 115, 710
		L. longbeachae	NA	NA
		L. sainthelensi	NA	NA
	19	L. feeleii	NA	NA
		L. anisa	NA	NA
December 2014	20	L. sainthelensi	NA	NA
	21	L. bozemanii	NA	NA
January 2015	22	L. pneumophila	1 (2)	84

a Species were typed by MALDI-TOF MS.

b NA, not applicable.

c X, a sequence type could not be retrieved due to failure of amplification of one or more gene targets.

d Legionella spp. were not further typed because the bacteria could not be recultured.

Positive samples were found throughout the year (see Table 1). The ambient mean daily temperatures on sampling days ranged from −1.9°C to 22.8°C (35). Positive garden soil samples were taken on days with ambient mean temperatures ranging between 0.7°C and 21.5°C. The precipitation sums of the 14 days preceding the sampling day varied between 0 and 113 mm for all sampling days and between 2 and 81 mm for the sampling days at which Legionella was detected.

### Resampling L. pneumophila-positive garden soils.

Six of the seven L. pneumophila-positive gardens were resampled after several months, and four again tested positive for Legionella (gardens 1, 4, 13, and 18; see Table 3). One of the L. pneumophila-positive gardens was not resampled because this garden was found to be positive at the end of the project. For three of the four gardens, L. pneumophila was again isolated in the second sampling (gardens 1, 13, 18). Garden 1 was resampled four times throughout the year and was found to be positive for L. pneumophila on all occasions. Several L. pneumophila STs were detected, with ST477 present on all four occasions and ST710 and ST84 present on three out of four occasions. L. longbeachae was also isolated on all 4 samplings of garden 1.

**TABLE 3:** Persistence of Legionella species in gardens 1, 4, 13, and 19, sampled on 2 to 4 occasions

Garden	Sampling parameters	First sampling	Second sampling	Third sampling	Fourth sampling
1	Sampling date (mo, yr)	February 2014	September 2014	November 2014	February 2015
	Detected species	L. pneumophila SG1	L. pneumophila SG1	L. pneumophila SG1	L. pneumophila SG1
		L. pneumophila SG2–14	L. pneumophila SG2–14	L. longbeachae	L. pneumophila SG2–14
		L. longbeachae	L. longbeachae		L. longbeachae
	Sequence types	84, 465, 477, 710, 863	84, 477, 710	84, 115, 477	477, 710
4	Sampling date (mo, yr)	April 2014	September 2014		
	Detected species	L. pneumophila SG1	L. feeleii		
		L. pneumophila SG2–14	L. sainthelensi		
		L. bozemanii			
		L. feeleii			
	Sequence type(s)	84, 115, 477, 863, 2028, 2032, X^*a*^	NA^*b*^		
13	Sampling date (mo, yr)	August 2014	January 2015		
	Detected species	L. pneumophila SG2–14	L. pneumophila SG1		
		L. longbeachae	L. pneumophila SG2–14		
		L. sainthelensi	L. bozemanii		
			L. dumoffi		
	Sequence type(s)	710	84, 115, 710, 2080, X		
18	Sampling date (mo, yr)	November 2014	March 2015		
	Detected species	L. pneumophila SG1	L. pneumophila SG1		
		L. pneumophila SG2–14	L. longbeachae		
		L. longbeachae	L. feeleii		
		L. sainthelensi			
	Sequence types	84, 115, 477, 710, 1856, 2022, 2029	2022, X		

a X, a sequence type could not be retrieved due to failure of amplification of one or more gene targets.

b NA, not applicable.

### Clinical relevance of L. pneumophila isolates.

A total of 70 L. pneumophila isolates were typed by SBT. For five isolates, a ST could not be retrieved due to the failure of amplification of one or more gene targets. The remaining 65 isolates were classified into 15 different STs (see Table 4). According to the EWGLI SBT database (36) (accessed on 17 February 2016), eight of these STs (ST84, ST115, ST462, ST465, ST477, ST710, ST863, and ST1856) were previously isolated from patients. Some STs were found regularly in garden soils, namely, ST84, ST115, ST477, and ST710. ST84 was detected most often; of the seven L. pneumophila-positive gardens, ST84 was found in all but one. Of the L. pneumophila SG1 isolates, 16 were MAb 3/1 positive and 10 were MAb 3/1 negative (see Table S1 in the supplemental material, which shows all of the typing data of the L. pneumophila garden isolates).

**TABLE 4:** Isolated sequence types from garden soils and clinical relevance of the sequence types according to the EWGLI SBT database^*a*^

Sequence type	No. of gardens^*b*^	No. of patients^*c*^
84	6	13
115	4	8
710	4	2
477	3	4
863	2	1
462	1	1
465	1	2
1856	1	1
2022	1	0
2025	1	0
2026	1	0
2028	1	0
2029	1	0
2032	1	0
2080	1	0

a The database was accessed on 17 February 2016 (36).

b Number of gardens from which the sequence type was isolated.

c Number of patients from which the sequence type was isolated according to the EWGLI SBT database. A value of 0 indicates that no clinical strains were reported.

### Univariate and multivariate analyses.

The questionnaire covered the following variables: characteristics of the garden (type, size, location, age), use of potting soil/compost, origin of used potting soil/compost, use of other fertilizers (e.g., manure, artificial fertilizer), use of pesticides/herbicides, use of tap water for watering, presence of an outdoor whirlpool, season of sampling, and frequency of gardening in gardening season (see Table S2 in the supplemental material). The owners of all 177 sampled gardens filled in the questionnaire. In addition, eight weather variables were analyzed, namely, precipitation sum on the sampling day, precipitation sum in the 14 days preceding the sampling day, ambient temperature (minimum, mean, maximum) on the sampling day, and ambient temperature (minimum, mean, maximum) in the 14 days preceding the sampling day.

Six of the examined variables reached a *P* value of ≤0.25 in the univariate logistic regression and were analyzed in the multivariate analysis, i.e., garden size, garden age, use of potting soil/compost, use of other fertilizers, precipitation sum on the sampling day, and mean temperature in the 14 days preceding the sampling day (see Table S2 in the supplemental material). One variable (surrounding area) had a prevalence of zero in two out of three categories and could not be analyzed in the multivariate model. Correlations between all variables appeared to be low (i.e., *r* ≤ 0.25). Multivariate logistic regression analysis identified none of the variables to be statistically significantly associated with the presence of Legionella in the gardens.

## DISCUSSION

Viable Legionella strains, including L. pneumophila, were isolated by amoebal coculture from 12% of 177 investigated garden soils in the Netherlands, indicating that garden soil is a reservoir of Legionella bacteria. The majority of the isolated L. pneumophila SG1 strains were found to be MAb 3/1 positive. MAb 3/1 positivity is considered an indication of virulence since this monoclonal antibody recognizes a virulence-associated epitope (33). Eight STs found in garden soils were clinically relevant according to the EWGLI SBT database (36). However, the STs that are most often detected in patients in the Netherlands (ST1, ST47, and ST62) (16) were not detected in garden soils. Of the 15 detected STs in this study, three were also found in patients in the Netherlands (ST84, ST115, and ST477). However, these STs are relatively uncommon; they were found in three patients, four patients, and one patient, respectively (since 2005). Strikingly however, these three clinically relevant STs belonged to the most frequently isolated STs in garden samples (see Table 4). It is possible that some conditions in garden soils are favorable for the growth of these clinically relevant STs. So, although garden soil is probably not the source of most infections with L. pneumophila in the Netherlands, it should be taken into account that it may play a role in Legionella transmission to humans, which warrants further investigation.

L. sainthelensi was found most often in garden soil; it was isolated from 10 of the 22 positive gardens (see Tables 2 and 3). L. sainthelensi can be infectious to humans (37, 38) and was first isolated in the United States from fresh water (39). L. pneumophila was the second most isolated species, and L. longbeachae was the third. Strikingly, L. longbeachae was always detected in combination with L. pneumophila. Legionella bacteria were detected in garden soils throughout the year. No association was found between Legionella-positive gardens and temperature or precipitation. Evidence was found that L. pneumophila can persist in garden soil. For three gardens, L. pneumophila was detected again after 4 to 7 months, and for one garden, L. pneumophila was found on four sampling occasions over a period of 1 year.

Interestingly, Legionella was absent from garden soils in rural areas, whereas 14.6% of garden samples in urban areas were positive for Legionella (see Table S2 in the supplemental material, variable “surrounding area”). However, since only 9 gardens in rural areas were investigated, no firm conclusion can be drawn about the differences in Legionella presence in rural versus urban areas. Legionella was also absent from gardens in mixed areas (gardens located at the edge of a village, adjacent to a rural area).

It is possible that Legionella in garden soil originates from compost or potting soil. One Australian study, by Hughes and Steele (20), showed the presence of Legionella in six garden soils, of which five contained L. pneumophila. These soils were mixed with composted materials that were found to contain Legionella. Compost (20, 40, 41) and potting soil (42–44) are reservoirs of Legionella, and it is possible that Legionella is introduced into garden soil by the use of compost or potting soil in the garden. To investigate this possibility, genotypes isolated from garden soils can be compared to genotypes isolated from composts and potting soils. In this study, compost or potting soil samples were provided by the garden owners when available, but they were only analyzed when the compost/potting soil was ever applied to the area where the soil sample was taken and when the soil sample was found to be positive for L. pneumophila. For the seven L. pneumophila-positive gardens, only one potting soil sample was analyzed but not found to be positive. Furthermore, no association was found between Legionella-positive gardens and the reported use of potting soil or compost. We found only one publication that used SBT to type two L. pneumophila strains that were isolated from composted materials (40). Interestingly, these were ST84, the most frequently isolated ST in this study. In contrast to the limited number of garden soil studies, several studies have investigated natural soil as a reservoir of L. pneumophila (22–24, 27). No ST similarities were observed between the reported STs in these studies and the current study.

A limitation of the use of an amoebal coculture method for the isolation of Legionella is that selectivity for certain Legionella strains might be introduced. It is possible that certain STs replicate better in A. castellanii than other STs, resulting in an outcome that is not representative of the natural situation. However, selectivity was not shown in a previous study in which a batch of 23 different L. pneumophila strains was tested (26). The batch was comprised of different monoclonal subtypes, both positive and negative for MAb 3/1, and 16 different STs. These strains, including the clinically relevant ST1 and ST47, were all shown to replicate similarly in amoebal coculture with A. castellanii. Another drawback of the amoebal coculture method is its limited sensitivity since only a small amount of soil is investigated per sample. However, for soil samples, amoebal coculture seems to be the best method in order to obtain isolates. In previous studies, we have shown that for samples containing many other bacteria, like soil, amoebal coculture has a higher positivity rate than culture techniques that are based solely on agar plates (26, 27). Another limitation of amoebal coculture is that only a restricted number of samples can be investigated since it is a rather laborious method. Therefore, only one sample per garden was analyzed, while multiple samples would probably have influenced the positivity rate. A more thorough investigation may have been done using molecular techniques, like PCR. However, molecular methods do not render isolates, which are necessary for typing purposes. Due to these limitations, including low sensitivity and the restricted number of samples, an accurate estimate of the true prevalence of Legionella in garden soils cannot be made. In order to study the prevalence, another sampling strategy and other analysis methods should be chosen.

The significance of the presence of Legionella in garden soils to public health is not clear. No patients are known to have been infected by Legionella originating from garden soil. In several older publications, natural soil was considered to be a possible source of infection (25, 45–49) because excavation sites were believed to be associated with LD cases (50, 51). However, these studies provided no or little evidence for soil as a source of Legionella (9). One more recent study provided evidence for infection caused by L. pneumophila originating from natural soil (21). The infective strain was isolated from a field where the LD case had spent several days potting plants.

In conclusion, garden soil is a reservoir of L. pneumophila and Legionella spp. and may be an alternative source of Legionella that is not considered in source investigations, especially for some soil-specific strains like ST84, ST115, and ST477. Whether the presence of Legionella in garden soil has an impact on public health is not clear; no cases are linked to Legionella in garden soil, and none of the most prevalent Dutch clinical strains were identified in garden soil in this study. A case-control study may reveal whether gardening or working with garden soil is a risk factor for contracting LD, warranting targeted interventions. Prevalence should be studied in more detail, and Legionella concentrations in garden soil should be determined. Furthermore, it should be investigated how soil and other environmental conditions, i.e., weather characteristics, influence viability, growth, and virulence of Legionella in garden soil. Furthermore, it is important to investigate if and how Legionella bacteria can aerosolize from soil and which gardening activities might pose a risk.

## Supplementary Material

Supplemental material

## References

[1] Fields BS, Benson RF, Besser RE 2002 Legionella and Legionnaires' disease: 25 years of investigation. Clin Microbiol Rev 15:506–526. doi:10.1128/CMR.15.3.506-526.2002. 12097254 PMC118082

[2] Beaute J, Zucs P, de Jong B, European Legionnaires' Disease Surveillance Network 2013 Legionnaires disease in Europe, 2009-2010. Euro Surveill 18(10):pii=20417 http://www.eurosurveillance.org/ViewArticle.aspx?ArticleId=20417. 10.2807/ese.18.10.20417-en 23515061

[3] Joseph CA, Ricketts KD, European Working Group for Legionella Infections 2010 Legionnaires disease in Europe 2007-2008. Euro Surveill 15(8):pii=19493 http://www.eurosurveillance.org/ViewArticle.aspx?ArticleId=19493. 20197022

[4] Ricketts KD, Joseph CA, European Working Group for Legionella Infections 2007 Legionnaires disease in Europe: 2005-2006. Euro Surveill 12(12):pii=753 http://www.eurosurveillance.org/ViewArticle.aspx?ArticleId=753.

[5] Benin AL, Benson RF, Besser RE 2002 Trends in Legionnaires disease, 1980-1998: declining mortality and new patterns of diagnosis. Clin Infect Dis 35:1039–1046. doi:10.1086/342903. 12384836

[6] NNDSS Annual Report Writing Group. 2015 Australia's notifiable disease status, 2012: annual report of the National Notifiable Diseases Surveillance System. Commun Dis Intell Q Rep 39:E46–E136. 26063098 10.33321/cdi.2015.39.7

[7] Phares CR, Wangroongsarb P, Chantra S, Paveenkitiporn W, Tondella ML, Benson RF, Thacker WL, Fields BS, Moore MR, Fischer J, Dowell SF, Olsen SJ 2007 Epidemiology of severe pneumonia caused by Legionella longbeachae, Mycoplasma pneumoniae, and Chlamydia pneumoniae: 1-year, population-based surveillance for severe pneumonia in Thailand. Clin Infect Dis 45:e147–e155. doi:10.1086/523003. 18190309

[8] Graham FF, White PS, Harte DJ, Kingham SP 2012 Changing epidemiological trends of legionellosis in New Zealand, 1979-2009. Epidemiol Infect 140:1481–1496. doi:10.1017/S0950268811000975. 21943591

[9] van Heijnsbergen E, Schalk JA, Euser SM, Brandsema PS, den Boer JW, de Roda Husman AM 2015 Confirmed and potential sources of Legionella reviewed. Environ Sci Technol 49:4797–4815. doi:10.1021/acs.est.5b00142. 25774976

[10] Walser SM, Gerstner DG, Brenner B, Holler C, Liebl B, Herr CE 2014 Assessing the environmental health relevance of cooling towers–a systematic review of legionellosis outbreaks. Int J Hyg Environ Health 217:145–154. doi:10.1016/j.ijheh.2013.08.002. 24100053

[11] Coetzee N, Duggal H, Hawker J, Ibbotson S, Harrison TG, Phin N, Laza-Stanca V, Johnston R, Iqbal Z, Rehman Y, Knapper E, Robinson S, Aigbogun N 2012 An outbreak of Legionnaires' disease associated with a display spa pool in retail premises, Stoke-on-Trent, United Kingdom, July 2012. Euro Surveill 17(37):pii=20271 http://www.eurosurveillance.org/ViewArticle.aspx?ArticleId=20271. 22995431

[12] Miyamoto H, Jitsurong S, Shiota R, Maruta K, Yoshida S, Yabuuchi E 1997 Molecular determination of infection source of a sporadic Legionella pneumonia case associated with a hot spring bath. Microbiol Immunol 41:197–202. doi:10.1111/j.1348-0421.1997.tb01190.x. 9130230

[13] Borgen K, Aaberge I, Werner-Johansen O, Gjosund K, Storsrud B, Haugsten S, Nygard K, Krogh T, Hoiby EA, Caugant DA, Kanestrom A, Simonsen O, Blystad H 2008 A cluster of Legionnaires' disease linked to an industrial plant in southeast Norway, June-July 2008. Euro Surveill 13(38):pii=18985 http://www.eurosurveillance.org/ViewArticle.aspx?ArticleId=18985. 18801321

[14] Lindsay DSJ, Brown AW, Brown DJ, Pravinkumar SJ, Anderson E, Edwards GFS 2012 Legionella longbeachae serogroup 1 infections linked to potting compost. J Med Microbiol 61:218–222. doi:10.1099/jmm.0.035857-0. 21940651

[15] Pravinkumar SJ, Edwards G, Lindsay D, Redmond S, Stirling J, House R, Kerr J, Anderson E, Breen D, Blatchford O, McDonald E, Brown A 2010 A cluster of Legionnaires' disease caused by Legionella longbeachae linked to potting compost in Scotland, 2008-2009. Euro Surveill 15(8):pii=19496 http://www.eurosurveillance.org/ViewArticle.aspx?ArticleId=19496. 10.2807/ese.15.08.19496-en 20197024

[16] Den Boer JW, Euser SM, Brandsema P, Reijnen L, Bruin JP 2015 Results from the National Legionella Outbreak Detection Program, the Netherlands, 2002-2012. Emerg Infect Dis 21:1167–1173. doi:10.3201/eid2107.141130. 26079594 PMC4480379

[17] Euser SM, Bruin JP, Brandsema P, Reijnen L, Boers SA, Den Boer JW 2013 Legionella prevention in the Netherlands: an evaluation using genotype distribution. Eur J Clin Microbiol Infect Dis 32:1017–1022. doi:10.1007/s10096-013-1841-9. 23430195

[18] Harrison TG, Afshar B, Doshi N, Fry NK, Lee JV 2009 Distribution of Legionella pneumophila serogroups, monoclonal antibody subgroups and DNA sequence types in recent clinical and environmental isolates from England and Wales (2000-2008). Eur J Clin Microbiol Infect Dis 28:781–791. doi:10.1007/s10096-009-0705-9. 19156453

[19] Schalk JA, Euser SM, van Heijnsbergen E, Bruin JP, den Boer JW, de Roda Husman AM 2014 Soil as a source of Legionella pneumophila sequence type 47. Int J Infect Dis 27:18–19. doi:10.1016/j.ijid.2014.05.009. 25130616

[20] Hughes MS, Steele TW 1994 Occurrence and distribution of Legionella species in composted plant materials. Appl Environ Microbiol 60:2003–2005. 11001749 10.1128/aem.60.6.2003-2005.1994 PMC201593

[21] Wallis L, Robinson P 2005 Soil as a source of Legionella pneumophila serogroup 1 (Lp1). Aust N Z J Public Health 29:518–520. doi:10.1111/j.1467-842X.2005.tb00242.x. 16366061

[22] Travis TC, Brown EW, Peruski LF, Siludjai D, Jorakate P, Salika P, Yang G, Kozak NA, Kodani M, Warner AK, Lucas CE, Thurman KA, Winchell JM, Thamthitiwat S, Fields BS 2012 Survey of Legionella species found in Thai soil. Int J Microbiol 2012:218791. 22287969 10.1155/2012/218791 PMC3263619

[23] Amemura-Maekawa J, Kikukawa K, Helbig JH, Kaneko S, Suzuki-Hashimoto A, Furuhata K, Chang B, Murai M, Ichinose M, Ohnishi M, Kura F, Working Group for Legionella in Japan 2012 Distribution of monoclonal antibody subgroups and sequence-based types among Legionella pneumophila serogroup 1 isolates derived from cooling tower water, bathwater, and soil in Japan. Appl Environ Microbiol 78:4263–4270. doi:10.1128/AEM.06869-11. 22492442 PMC3370504

[24] Kuroki H, Miyamoto H, Fukuda K, Iihara H, Kawamura Y, Ogawa M, Wang Y, Ezaki T, Taniguchi H 2007 Legionella impletisoli sp. nov. and Legionella yabuuchiae sp. nov., isolated from soils contaminated with industrial wastes in Japan. Syst Appl Microbiol 30:273–279. doi:10.1016/j.syapm.2006.11.005. 17197147

[25] Cordes LG, Fraser DW, Skaliy P, Perlino CA, Elsea WR, Mallison GF, Hayes PS 1980 Legionnaires' disease outbreak at an Atlanta, Georgia, country club: evidence for spread from an evaporative condenser. Am J Epidemiol 111:425–431. 7377185 10.1093/oxfordjournals.aje.a112917

[26] Schalk JA, Docters van Leeuwen AE, Lodder WJ, de Man H, Euser S, den Boer JW, de Roda Husman AM 2012 Isolation of Legionella pneumophila from pluvial floods by amoebal coculture. Appl Environ Microbiol 78:4519–4521. doi:10.1128/AEM.00131-12. 22467504 PMC3370508

[27] van Heijnsbergen E, de Roda Husman AM, Lodder WJ, Bouwknegt M, Docters van Leeuwen AE, Bruin JP, Euser SM, den Boer JW, Schalk JA 2014 Viable Legionella pneumophila bacteria in natural soil and rainwater puddles. J Appl Microbiol 117:882–890. doi:10.1111/jam.12559. 24888231

[28] Rowbotham TJ 1983 Isolation of Legionella pneumophila from clinical specimens via amoebae, and the interaction of those and other isolates with amoebae. J Clin Pathol 36:978–986. doi:10.1136/jcp.36.9.978. 6350372 PMC498455

[29] Hayden RT, Uhl JR, Qian X, Hopkins MK, Aubry MC, Limper AH, Lloyd RV, Cockerill FR 2001 Direct detection of Legionella species from bronchoalveolar lavage and open lung biopsy specimens: comparison of LightCycler PCR, *in situ* hybridization, direct fluorescence antigen detection, and culture. J Clin Microbiol 39:2618–2626. doi:10.1128/JCM.39.7.2618-2626.2001. 11427579 PMC88195

[30] Gaia V, Fry NK, Afshar B, Luck PC, Meugnier H, Etienne J, Peduzzi R, Harrison TG 2005 Consensus sequence-based scheme for epidemiological typing of clinical and environmental isolates of Legionella pneumophila. J Clin Microbiol 43:2047–2052. doi:10.1128/JCM.43.5.2047-2052.2005. 15872220 PMC1153775

[31] Ratzow S, Gaia V, Helbig JH, Fry NK, Luck PC 2007 Addition of *neuA*, the gene encoding *N*-acylneuraminate cytidylyl transferase, increases the discriminatory ability of the consensus sequence-based scheme for typing Legionella pneumophila serogroup 1 strains. J Clin Microbiol 45:1965–1968. doi:10.1128/JCM.00261-07. 17409215 PMC1933043

[32] Boers SA, van der Reijden WA, Jansen R 2012 High-throughput multilocus sequence typing: bringing molecular typing to the next level. PLoS One 7:e39630. doi:10.1371/journal.pone.0039630. 22815712 PMC3399827

[33] Helbig JH, Bernander S, Castellani Pastoris M, Etienne J, Gaia V, Lauwers S, Lindsay D, Luck PC, Marques T, Mentula S, Peeters MF, Pelaz C, Struelens M, Uldum SA, Wewalka G, Harrison TG 2002 Pan-European study on culture-proven Legionnaires' disease: distribution of Legionella pneumophila serogroups and monoclonal subgroups. Eur J Clin Microbiol Infect Dis 21:710–716. doi:10.1007/s10096-002-0820-3. 12415469

[34] Hosmer DW, Lemeshow S 1989 Applied logistic regression. Wiley, New York, NY.

[35] KNMI. 2015 Daily meteorological data in the Netherlands. KNMI, De Bilt, the Netherlands http://knmi.nl/nederland-nu/klimatologie/daggegevens.

[36] EWGLI. 2016 Sequence-based typing database for Legionella pneumophila. http://www.hpa-bioinformatics.org.uk/legionella/legionella_sbt/php/sbt_homepage.php Accessed 17 February 2016.

[37] Loeb M, Simor AE, Mandell L, Krueger P, McArthur M, James M, Walter S, Richardson E, Lingley M, Stout J, Stronach D, McGeer A 1999 Two nursing home outbreaks of respiratory infection with Legionella sainthelensi. J Am Geriatr Soc 47:547–552. doi:10.1111/j.1532-5415.1999.tb02568.x. 10323647 PMC7166437

[38] Han XY, Ihegword A, Evans SE, Zhang J, Li L, Cao H, Tarrand JJ, El-Kweifi O 2015 Microbiological and clinical studies of legionellosis in 33 patients with cancer. J Clin Microbiol 53:2180–2187. doi:10.1128/JCM.00380-15. 25926494 PMC4473214

[39] Campbell J, Bibb WF, Lambert MA, Eng S, Steigerwalt AG, Allard J, Moss CW, Brenner DJ 1984 Legionella sainthelensi: a new species of Legionella isolated from water near Mt. St Helens. Appl Environ Microbiol 47:369–373. 6712210 10.1128/aem.47.2.369-373.1984 PMC239676

[40] McCabe S, Brown A, Edwards GFS, Lindsay D 2011 Enhanced isolation of Legionella species from composted material. Clin Microbiol Infect 17:1517–1520. doi:10.1111/j.1469-0691.2011.03582.x. 21848971

[41] Casati S, Conza L, Bruin J, Gaia V 2010 Compost facilities as a reservoir of Legionella pneumophila and other Legionella species. Clin Microbiol Infect 16:945–947. doi:10.1111/j.1469-0691.2009.03009.x. 19645762

[42] Casati S, Gioria-Martinoni A, Gaia V 2009 Commercial potting soils as an alternative infection source of Legionella pneumophila and other Legionella species in Switzerland. Clin Microbiol Infect 15:571–575. doi:10.1111/j.1469-0691.2009.02742.x. 19392903

[43] Koide M, Arakaki N, Saito A 2001 Distribution of Legionella longbeachae and other legionellae in Japanese potting soils. J Infect Chemother 7:224–227. doi:10.1007/s101560170017. 11810588

[44] Steele TW, Lanser J, Sangster N 1990 Isolation of Legionella longbeachae serogroup 1 from potting mixes. Appl Environ Microbiol 56:49–53. 1968736 10.1128/aem.56.1.49-53.1990 PMC183249

[45] Parry MF, Stampleman L, Hutchinson JH, Folta D, Steinberg MG, Krasnogor LJ 1985 Waterborne Legionella bozemanii and nosocomial pneumonia in immunosuppressed patients. Ann Intern Med 103:205–210. doi:10.7326/0003-4819-103-2-205. 4014902

[46] Eng RH, Rothkopf M, Smith SM, Shah Y, Perez E, McDearman SC 1984 Legionnaires' disease in a gravedigger. An epidemiologic study. N Y State J Med 84:238–240. 6588320

[47] Conwill DE, Werner SB, Dritz SK, Bissett M, Coffey E, Nygaard G, Bradford L, Morrison FR, Knight MW 1982 Legionellosis—the 1980 San Francisco outbreak. Am Rev Respir Dis 126:666–669. 7125358 10.1164/arrd.1982.126.4.666

[48] Morris GK, Patton CM, Feeley JC, Johnson SE, Gorman G, Martin WT, Skaliy P, Mallison GF, Politi BD, Mackel DC 1979 Isolation of the Legionnaires' disease bacterium from environmental samples. Ann Intern Med 90:664–666. doi:10.7326/0003-4819-90-4-664. 373549

[49] Haley CE, Cohen ML, Halter J, Meyer RD 1979 Nosocomial Legionnaires' disease: a continuing common-source epidemic at Wadsworth Medical Center. Ann Intern Med 90:583–586. doi:10.7326/0003-4819-90-4-583. 373547

[50] Storch G, Baine WB, Fraser DW, Broome CV, Clegg HW II, Cohen ML, Goings SA, Politi BD, Terranova WA, Tsai TF, Plikaytis BD, Shepard CC, Bennett JV 1979 Sporadic community-acquired Legionnaires' disease in the United States. A case-control study. Ann Intern Med 90:596–600. 434642 10.7326/0003-4819-90-4-596

[51] Thacker SB, Bennett JV, Tsai TF, Fraser DW, McDade JE, Shepard CC, Williams KH Jr, Stuart WH, Dull HB, Eickhoff TC 1978 An outbreak in 1965 of severe respiratory illness caused by the Legionnaires' disease bacterium. J Infect Dis 138:512–519. doi:10.1093/infdis/138.4.512. 361897

