# Supplementary material for: Presence and Persistence of Viable, Clinically Relevant Legionella pneumophila Bacteria in Garden Soil in the Netherlands
Source: Appl Environ Microbiol. 2016 Aug 15;82(17):5125–31. doi: 10.1128/AEM.00595-16 (PMC4988192; doi:10.1128/AEM.00595-16)
Supplement: Supplemental material [file supp_82_17_5125__index.html]

Supplemental material 

# Presence and Persistence of Viable, Clinically Relevant Legionella pneumophila Bacteria in Garden Soil in the Netherlands

## Supplemental material

- Supplemental file 1 -

  All typing data of the *L. pneumophila* garden isolates (Table S1); all variables univariately analyzed for association with the presence of *Legionella* bacteria in garden soils (Table S2).

  PDF, 153K
