# Supplementary material for: Presence and Persistence of Viable, Clinically Relevant Legionella pneumophila Bacteria in Garden Soil in the Netherlands
Source: Appl Environ Microbiol. 2016 Aug 15;82(17):5125–31. doi: 10.1128/AEM.00595-16 (PMC4988192; doi:10.1128/AEM.00595-16)
Supplement: Supplemental material [file AEM.00595-16_zam999117363so1.pdf]

Supplemental material

Table S1. Typing results of the *L. pneumophila* strains isolated from garden soil samples. ST; sequence type, X; a sequence type could not be retrieved due to failure of amplification of one or more gene targets.

Garden	Sampling	Sampling yr	Sampling mo	Serogroup	Mab-3/1 positive/negative	Mab-subtype	ST	No. of typed strains
**1**	1st	2014	February	SG1	pos	Benidorm	84	2
**1**	1st	2014	February	SG1	pos	Benidorm	477	4
**1**	1st	2014	February	SG1	pos	France/Allentown	863	1
**1**	1st	2014	February	SG 2-14	na	na	465	1
**1**	1st	2014	February	SG 2-14	na	na	710	1
**1**	2nd	2014	September	SG1	pos	Benidorm	84	2
**1**	2nd	2014	September	SG1	pos	Benidorm	477	1
**1**	2nd	2014	September	SG 2-14	na	na	710	3
**1**	3rd	2014	November	SG1	pos	Benidorm	84	1
**1**	3rd	2014	November	SG1	pos	Benidorm	115	2
**1**	3rd	2014	November	SG1	pos	Benidorm	477	3
**1**	4th	2015	February	SG1	neg	Camperdown	477	4
**1**	4th	2015	February	SG 2-14	na	na	710	2
**4**	1st	2014	April	SG1	pos	Benidorm	84	1
**4**	1st	2014	April	SG1	pos	Benidorm	115	2
**4**	1st	2014	April	SG1	pos	Benidorm	477	1
**4**	1st	2014	April	SG1	pos	Philadelphia	2032	1
**4**	1st	2014	April	SG1	neg	Camperdown	2028	1
**4**	1st	2014	April	SG 2-14	na	na	863	1
**4**	1st	2014	April	SG 2-14	na	na	X	2
**5**	1st	2014	April	SG 2-14	na	na	2025	2
**5**	1st	2014	April	SG 2-14	na	na	2026	1
**5**	1st	2014	April	SG 2-14	na	na	X	1
**8**	1st	2014	June	SG1	pos	Benidorm	84	1
**8**	1st	2014	June	SG1	pos	Benidorm	710	1
**8**	1st	2014	June	SG 2-14	na	na	462	1
**13**	1st	2014	August	SG 2-14	na	na	710	2
**13**	2nd	2015	January	SG1	neg	Camperdown	84	5
**13**	2nd	2015	January	SG1	neg	Camperdown	115	1
**13**	2nd	2015	January	SG 2-14	na	na	84	1
**13**	2nd	2015	January	SG 2-14	na	na	710	1
**13**	2nd	2015	January	SG 2-14	na	na	2080	1
**13**	2nd	2015	January	SG 2-14	na	na	X	1
**18**	1st	2014	November	SG1	pos	Benidorm	84	1
**18**	1st	2014	November	SG1	pos	Benidorm	477	3
**18**	1st	2014	November	SG1	neg	Camperdown	1856	1
**18**	1st	2014	November	SG1	neg	Olda	1856	1
**18**	1st	2014	November	SG1	neg	Olda	2022	1
**18**	1st	2014	November	SG1	neg	Olda	2029	1
**18**	1st	2014	November	SG 2-14	na	na	84	1
**18**	1st	2014	November	SG 2-14	na	na	115	1
**18**	1st	2014	November	SG 2-14	na	na	710	1
**18**	2nd	2015	March	SG1	neg	Camperdown	X	1
**18**	2nd	2015	March	SG1	neg	Olda	2022	1
**22**	1st	2015	January	SG 2-14	na	na	84	2

Table S2. Questionnaire and weather variables univariately analyzed for association with the presence of *Legionella* in garden soils. A total of 177 gardens were sampled, if the total per variable does not tally to 177 then the variable contained missing data. Variables in bold were analyzed multivariately (P ≤ 0.25).

Variable		Frequency (no.)	Frequency (%)	Positive for *Legionella* (%)	*P-value*
Garden type	normal garden	164	92.7	12.2	0.74
	vegetable garden	7	4.0	28.6	
	allotment garden	6	3.4	0	
**Size garden**	≤30 m^2	82	46.3	15.9	0.17
	>30 m^2	80	45.2	8.8	
Surrounding area	rural	9	5.1	0	0.02
	urban	144	81.4	14.6	
	mixed	17	9.6	0	
**Age garden**	<1 year	10	6.4	30	0.10
	>1 year	146	93.6	10.3	
**Use potting soil/compost**	sometimes/regularly (at least 1/year)	103	59.9	14.6	0.24
	never	69	40.1	8.7	
Use of potting soil and origin potting soil/compost	yes, homemade	12	6.8	0	0.33
	yes, bought	77	43.5	16.9	
	yes, homemade and bought	8	4.5	12.5	
	yes, unknown	1	0.6	0	
	no, no use of potting soil/compost	69	39.0	8.7	
	yes, other	5	2.8	20.0	
**Use of other fertilizers**	sometimes/regularly (at least 1/year)	77	44.8	14.3	0.23
	never	95	55.2	8.4	
Frequency of gardening in gardening season	1/week or more	99	57.2	11.1	0.82
	1/month	40	23.1	15.0	
	<1/month	34	19.7	11.8	
Watering garden with tapwater	yes	133	75.1	12.8	0.67
	no	39	22.0	10.3	
Whirlpool in garden	yes	3	1.7	0	0.37
	no	174	98.3	12.6	
Use of pesticide/herbicide	yes	36	20.8	13.9	0.72
	no	137	79.2	11.7	

Table S2 (continued)

Variable		Frequency (no.)	Frequency (%)	Positive for *Legionella* (%)	*P-value*
Season of sampling	winter	37	20.9	13.5	0.89
	spring	47	26.6	10.6	
	summer	46	26.0	15.2	
	fall	47	26.6	10.6	
**Precipitation on the day of sampling (mm)**	0	71	40.1	16.9	0.22
	>0 - 1	19	10.7	10.5	
	>1 - 2	42	23.7	7.1	
	>2	45	25.4	11.1	
Precipitation sum in the 14 days preceding sampling (mm)	≤15	38	21.5	10.5	0.37
	>15 - 30	59	33.3	18.6	
	>30 - 45	31	17.5	9.7	
	>45	49	27.7	8.2	
Mean temperature on sampling day (°C)	≤6	41	23.2	12.2	0.56
	>6 - 12	27	15.3	11.1	
	>12 - 18	51	28.8	17.7	
	>18	58	32.8	8.6	
Minimum temperature on sampling day (°C)	≤4	44	24.9	13.6	0.93
	>4 - 9	39	22.0	10.3	
	>9 - 14	57	32.2	14.0	
	>14	37	20.9	10.8	
Maximum temperature on sampling day (°C)	≤11	50	28.2	12.0	0.70
	>11 - 16	25	14.1	8.0	
	>16 - 21	40	22.6	17.5	
	>21	62	35.0	11.3	
**Mean temperature in 14 days preceding sampling day (°C)**	≤8	39	22.0	15.4	0.16
	>8 - 12	46	26.0	13.0	
	>12 - 16	47	26.6	4.3	
	>16	45	25.4	17.8	
Minimum temperature in 14 days preceding sampling day (°C)	≤-1	37	20.9	10.8	0.58
	>-1 - 3	49	27.7	16.3	
	>3 - 7	41	23.2	7.3	
	>7	50	28.2	14.0	
Maximum temperature in 14 days preceding sampling day (°C)	≤17	49	27.7	16.3	0.68
	>17 - 22	37	20.9	10.8	
	>22 - 27	47	26.6	8.5	
	>27	44	24.9	13.6	
